# Spatial Distribution and Biodiversity of *Anopheles* Mosquito Species Across Climatic Zones in Burkina Faso: Implications for Malaria Vector Control

**DOI:** 10.3390/tropicalmed11010001

**Published:** 2025-12-19

**Authors:** Odette N. Zongo, Emmanuel Kiendrebeogo, Bazoumana B. D. Sow, Mahamadi Kientega, Inoussa Toé, Roger Sanou, Saberé O. G. Yemien, Grégoire Sawadogo, Honorine Kaboré, Achaz Agolinou, Nouhoun Traore, Patric Stephane Epopa, Abdoul Azize Millogo, Abdoulaye Niang, Moussa Namountougou, Hamidou Maiga, Abdoulaye Diabaté

**Affiliations:** 1 Institut de Recherche en Sciences de la Santé (IRSS)/Direction Régionale de l’Ouest (IRSS-DRO), Bobo-Dioulasso 01 BP 545, Burkina Faso; emmanuelkiendrebeogo0@gmail.com (E.K.); sowbazoumana@gmail.com (B.B.D.S.); mkient54@gmail.com (M.K.); inoussatoe11@gmail.com (I.T.); sanourog@yahoo.fr (R.S.); gillesyemien@gmail.com (S.O.G.Y.); gregsawadogo99@gmail.com (G.S.); honorine_kabore@yahoo.com (H.K.); agolinouachaz@gmail.com (A.A.); nouhoun89@gmail.com (N.T.); epopastef@yahoo.fr (P.S.E.); azizemillogo@gmail.com (A.A.M.); bband79@yahoo.fr (A.N.); npiediab@gmail.com (A.D.); 2 Unité de Formation et de Recherche en Sciences de la Vie et de la Terre, Université Nazi Boni, Bobo-Dioulasso 01 BP 1091, Burkina Faso; namountougou_d@yahoo.fr; 3 Institut des Sciences des Sociétés, Ouagadougou 03 BP 7047, Burkina Faso

**Keywords:** *Anopheles* species, biodiversity indices, climatic zones, malaria, vector control strategies

## Abstract

Malaria transmission in sub-Saharan Africa is dominated by the *An. gambiae* complex and *An. funestus* group, whose distribution varies across ecological settings. Secondary species occur at lower densities, but their role in transmission may differ from one locality to another depending on local conditions. Assessing *Anopheles* biodiversity using ecological indices is therefore essential to characterise their diversity and relative abundance. This study investigated the biodiversity and spatial distribution of *Anopheles* species across the three climatic zones of Burkina Faso to guide effective vector control strategies. Indoor resting mosquitoes were collected from 67 health districts across the 13 regions of Burkina Faso between September and December 2022 using pyrethroid spray catches. A total of 30,521 *Anopheles* mosquitoes were identified, with *An. gambiae s.l.* dominating (94.4%). The Sudano-Sahelian zone recorded the highest abundance, followed by the Soudanian and Sahelian zones. Biodiversity decreased from humid southern to arid northern areas, with the Soudanian zone showing the highest diversity. Molecular analysis of 2026 *An. gambiae s.l.* specimens revealed marked heterogeneity: *An. coluzzii* predominated in Sahelian (74.9%) and Sudano-Sahelian (71.2%) zones, while *An. gambiae s.s.* was most frequent in the Soudanian zone (53.8%). These results highlight spatial and ecological differences in *Anopheles* composition across Burkina Faso and emphasize the need for locally adapted malaria vector control strategies.

## 1. Introduction

Over the past two decades, substantial progress has been achieved in malaria control, especially through the large-scale implementation of preventive and therapeutic interventions [1,2]. Vector control strategies such as large distribution and use of insecticide-treated nets (ITNs), and indoor residual spraying (IRS), have played a central role in malaria control. These interventions are credited with over 70% of the substantial decline in malaria cases during this period [1]. Despite these achievements, malaria remains a major public health concern in sub-Saharan Africa [3], accounting for over 263 million reported cases and nearly 600,000 deaths in 2023 according to the WHO. This persistence is driven mainly by the emergence of drug resistance in *Plasmodium* species, insecticide resistance in mosquito populations, and behavioural changes that undermine the effectiveness of existing control tools [4,5]. The disease is caused by five *Plasmodium* species, with *P. falciparum* being the most prevalent and virulent in Africa [6]. Transmission occurs through female *Anopheles* mosquitoes, a genus comprising nearly 500 species worldwide, of which about 100 species are recognised as malaria vectors [7]. 

Mosquitoes (*Culicidae*) are widely distributed across tropical and temperate regions, comprising more than 3500 species grouped into three subfamilies. Among these, *Anopheles* species are of major medical importance as malaria vectors [8,9]. Africa hosts a wide diversity of *Anopheles* mosquitoes, with hundreds of species described across the continent [10]. However, only a subset is responsible for most malaria parasites transmission, and their distribution varies across ecological zones. In sub–Saharan Africa, the dominant vectors are *An. gambiae s.s.*, *An. coluzzii*, *An. arabiensis*, and *An. funestus* [11]. Other species such as *An. nili*, *An. rufipes*, *An. pharoensis*, and *An. coustani* typically occur at lower densities and act as secondary or potential vectors depending on local ecological conditions. The importance of these species varies across regions, reflecting differences in climate, vegetation, and human activities [12]. 

In Burkina Faso, the main malaria vectors are also *An. gambiae s.s.*, *An. coluzzii*, *An. arabiensis*, and *An. funestus* [13,14]. Ecological and climatic factors influence their distribution. *Anopheles gambiae s.s.* is typically associated with temporary, rain-dependent breeding sites, whereas *An. coluzzii* predominates in irrigated and semi-permanent habitats such as rice fields; *An. arabiensis* is now present in urban areas, probably reflecting its adaptation to human-modified environments [15]. In addition to these dominant vectors, several secondary *Anopheles* species, including *An. nili*, *An. pharoensis*, *An. rufipes*, *An. coustani*, are also present. Their distribution varies across ecological zones and is shaped by climatic conditions, breeding site characteristics, and human activities such as irrigation and urbanisation. However, the invasive *An. stephensi* has not yet been detected.

Human-driven environmental changes, such as urbanisation, deforestation, and agricultural expansion, influence mosquito ecology [16]. These alterations modify habitat availability, species composition, and population abundance, which can increase in vector species adapted to those altered ecosystems. Consequently, human vector contact may increase, thereby affecting the transmission dynamics of mosquito-borne diseases, including malaria [16]. To better understand how these environmental changes influence vector populations and transmission risk, biodiversity indices were used to provide reliable quantitative tools for describing and comparing mosquito populations across spatial and temporal gradients. Species richness (S) indicates the number of species present, the Shannon index (H′) integrates both richness and evenness, while the Simpson index (D) reflects dominance patterns within the populations. By combining information on species richness and relative abundance, these indices offer valuable insights into community structure and help elucidate the ecological complexity underlying malaria transmission systems. Applying such metrics to *Anopheles* populations enhances the understanding of vector diversity and informs adaptive, evidence-based control strategies [17]. Several studies have examined the diversity, distribution, and ecology of mosquitoes in different regions of Burkina Faso; however, most have focused primarily on malaria vectors and were geographically limited [9]. 

The present study provides an updated, nationwide assessment of *Anopheles* species diversity across all the climatic zones and area of the country, combining morphological and molecular identification methods with biodiversity indices. Understanding mosquito biodiversity within these changing environments is essential for designing effective surveillance and control strategies, which rely on accurate identification and characterisation of vector species across ecological contexts. By exploring spatial and ecological variations in vector composition, this work aims to enhance understanding of *Anopheles* biodiversity and support the development of more effective, locally adapted malaria control strategies.

## 2. Materials and Methods

### 2.1. Study Sites and Ecological Zones

**Figure 1:**
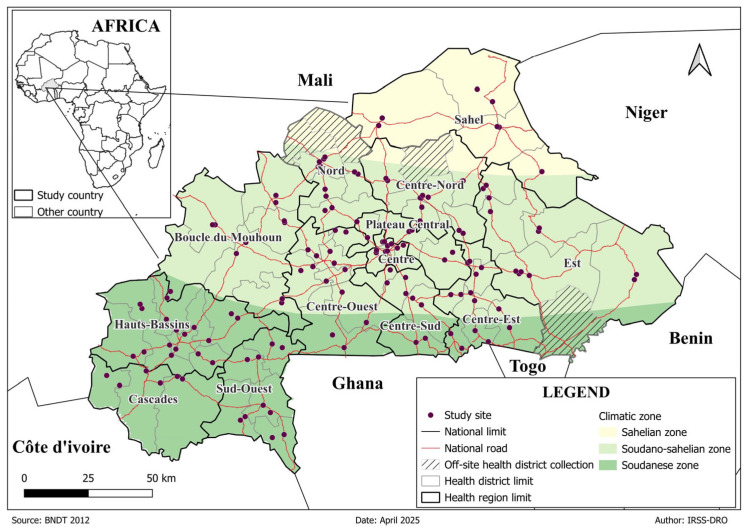
Spatial distribution of Health districts for *Anopheles* mosquito collection across Burkina Faso.

Entomological surveys were conducted in 67 of the 70 exiting health districts across the regions of Burkina Faso from September to December 2022. The country has a tropical climate with two distinct seasons: a rainy season lasting about 4 months (June–September) and a dry season of about 6 months (November–April). May and October being transition months. The country is divided into 70 health districts, organised around 13 administrative regions. These 13 regions are located in three main ecological zones: the Sahelian, Sudano-Sahelian, and Soudanian zones (Figure 1). Malaria control in these regions is based on the use of antimalarial drugs combined with preventive measures, including insecticide-based interventions and seasonal malaria chemoprevention for children under five years of age [18].

Each ecological zone is characterised by distinct climatic and environmental features that influence mosquito vector distribution. The Sahelian zone, located in the northern part of the country, represents the driest ecological region. It has a mean annual temperature of 29.1 °C and rainfall ranging from 300–600 mm per year, mostly falling between June and September. It is characterised by sparse vegetation, consisting mainly of grasslands, thorny shrubs, and drought-tolerant tree species. Agricultural activity is limited, with sorghum and millet as the dominant crops, typically cultivated near seasonal water bodies that may also serve as mosquito breeding habitats. The Sudano-Sahelian zone has a dry season from November to April, with average annual rainfall ranging between 600 and 900 mm and a mean annual temperature of 28.4 °C. Dry forests, savannas, and scattered trees dominate vegetation. Agricultural production is mainly based on cereal cultivation, particularly millet and maize, while vegetable farming is also carried out on a smaller scale. Furthermore, human activities such as vegetable cultivation and unregulated urban expansion contribute to the development of mosquito breeding habitats. The Soudanian zone, which receives the highest rainfall, 900–1200 mm annually, mainly from May to October, is rich in vegetation and constitutes the most humid ecological region in the country [19]. Gallery forests, dense savannas, and wooded landscapes dominate. Cotton and cereal crops are widely cultivated, and the combination of abundant rainfall, lush vegetation, and standing water creates favourable ecological conditions for mosquito proliferation [20,21]. 

### 2.2. Mosquito and Metadata Collection

Indoor-resting mosquitoes were collected early in the morning (06:00–09:00 a.m.) using pyrethroid spray catches (PSC) from ten randomly selected houses per site across 67 health districts in Burkina Faso [22]. For each selected house, a single room was sprayed. The insecticide spray used for PSC was Kaltox Paalga^®^ (Saphyto, Bobo-Dioulasso, Burkina Faso), a commercially and locally used spray. Before spraying, food was moved outside. Inhabitants were also invited to move outside the house. A white sheet was spread on the floor of selected rooms, all openings were carefully closed, and the insecticide was sprayed in the room. After about ten minutes, all knocked-down mosquitoes were collected from the sheet, put in a Petri dish, carefully labelled and sent for the sample processing. Each collection site was georeferenced, and mosquito specimens were identified to the genus level. All specimens were counted, identified, and preserved in 80% ethanol for subsequent analyses. PCR assays were performed on *An. gambiae* complex (*s.l.*) specimens from 33 districts, selected based on the three main climatic zones, to determine species composition, specifically distinguishing *An. gambiae s.s.*, *An. coluzzii*, and *An. arabiensis*.

### 2.3. Molecular Identification of Anopheles gambiae s.l.

Molecular analyses were conducted to identify *An. gambiae* complex species. Genomic DNA was extracted from a single mosquito using a 2% CTAB protocol [23]. The extracted DNA served as the template for PCR amplification targeting the SINE200 region to identify species within the *An. gambiae* complex (*s.l.*). A single pair of primers, S200 X6.1 F (5′-TCGCCTTAGACCTTGCGTTA-3′) and S200 X6.1 R (5′-CGCTTCAAGAATTCGAGATAC-3′), was used with identification based on the size of the amplified fragment: approximately 479 bp for *An. coluzzii*, 249 bp for *An. gambiae s.s.*, and 223 bp for *An. arabiensis.* PCRs were carried out in a 20 μl reaction containing 3 µL of template DNA, 0.4 µL of each primer, 4 µL of 5 × Hot Firepol Blend Master Mix, and 12.2 µL of molecular-grade water. Thermocycler conditions were 95°C for 15 min followed by 35 cycles of denaturation at 95 °C for 1 min, annealing at 62°C for 1 min, extension at 72 °C for 1 min, final extension step at 72 °C for 5 min, and a 4 °C hold. PCR products were visualized on 2% agarose gels stained with ethidium bromide.

### 2.4. Data Management and Metadata

We managed our data in accordance with our approved data management plan (DMP), an approach was designed to uphold the FAIR principles, making data Findable, Accessible, Interoperable and Reusable, thereby guaranteeing its integrity and supporting reproducible results [24]. Field data were collected using the KoBoToolbox platform [25] on ruggedised tablets, with customised electronic forms incorporating validation rules to minimise entry errors. A two-step verification process [26] was applied, and the two resulting datasets were programmatically cross validated using the arsenal package [27], with discrepancies resolved by reference to original filed records. Missing data were handled according to predefined guidelines outlined in our DMP. For sporadic missing environmental covariates (<5%), multiple imputation by chained equation (MICE) was performed using the nice package [28] and results from five imputed datasets were pooled. The final validated dataset was archived in non-proprietary CSV format on secure, access-controlled servers with automated daily backups. In line with our commitment to data sharing, the de-identified dataset will be deposited in the Dryad Repository [29] upon manuscript acceptance, assigned a DOI and released under CCO waiver to ensure global accessibility and reusability.

### 2.5. Statistical Analyses

Statistical analyses were conducted in R version 4.5.1 to investigate mosquito species abundance, diversity patterns and their association with climatic zones and seasons. Species abundance and relative abundance were computed by aggregating counts of each *Anopheles* species by district and period. Relative abundance for each species was calculated as the proportion of its abundance to the total abundance within each district/period combination according to the following formula:

$${Relative\;Abundance}_{i}=\frac{N_{i}}{\sum_{j=1}^{S}N_{j}}$$

where Ni represents the abundance of species i and S denotes the total number of species in the population, which allows comparisons of species composition across spatial and temporal scales while accounting for variations in sampling effort [30].

Biodiversity metrics (species richness, Shannon diversity index and Simpson diversity index) were calculated to characterise population structure. Species richness (S) was quantified as the number of species with non-zero abundance in each district/period combination. The Shanon index (H’) was computed as

$$H\prime =-\sum_{i=1}^{S}p_{i}ln\left(p_{i}\right)$$

where pi represents the proportion of species *i* in the population. This index quantifies species diversity by combining richness and evenness with greater emphasis on rare species [31]. The Simpson index (D) was calculated as

$$D=1-\sum_{i=1}^{S}p_{i}^{2}$$

which represents the probability that two randomly selected individuals belong to the same species (range 0 < D ≤ 1), was calculated. For the primary analysis, D was transformed into a component, D’ = 1 − D, representing the probability that two randomly selected individuals range [0, 1]. This bounded metric (D) was selected for subsequent beta regression modeling. All diversity indices were computed using the “vegan version 2.7.1” package in R with appropriate handling of zero-inflated data through boundary adjustment (0.001 for zero and 0.999 for one) [32].

PCR-confirmed species data from *An. gambiae s.l*. specimens were used to validate morphological identifications and to assess species distribution patterns across climatic zones.

A contingency table of species counts by zone was built and chi-square tests of independence were performed to evaluate the association between climatic zone and species composition. The strength was quantified using Cramer’s V:

$$V=\sqrt{\frac{{chi}^{2}}{n\times min\left(r-1,c-1\right)}}$$

where chi^2^ denotes chi-square statistic, *n* represents the total sample size, and *r* and *c* correspond to dimensions of the contingency table [33]. Species proportions with 95% confidence intervals were calculated using the Clopper-Pearson exact method for binomial proportions [34] and visualised to highlight compositional differences among zones. 

To investigate the effects of climatic zone and season on biodiversity patterns, we used beta regression models with the Simpson index as the response variable. Given the bounded nature of diversity indices (0–1) and the presence of zero values, we implemented both standard beta regression and zero-inflated beta regression approaches using the “glmmTMB version 1.1.12” package [35]. Models were fitted with climatic zone and season as fixed effects and district as a random. Model selection was performed using the Akaike Information Criterion (AIC) to identify the most parsimonious model [24,36]. Separate models were fitted for each climatic zone to account for zone-specific dynamics, followed by a combined model including all zones to assess overall patterns.

In cases where frequentist models exhibited convergence issues or poor fit, we employed Bayesian beta regression models with the “brms version 2.23.0” package as an alternative [37]. These models were implemented with four chains, 2000 iterations (1000 warmup) and default weakly informative priors. Convergence was assessed using the R-hat statistic (target < 1.01) and effective sample sizes (target > 400) [38]. Posterior predictive checks were conducted to validate model fit, and summaries were reported with 95% credible intervals.

## 3. Results

### 3.1. Species Composition of Anopheles Mosquitoes Across Burkina Faso

**Figure 2:**
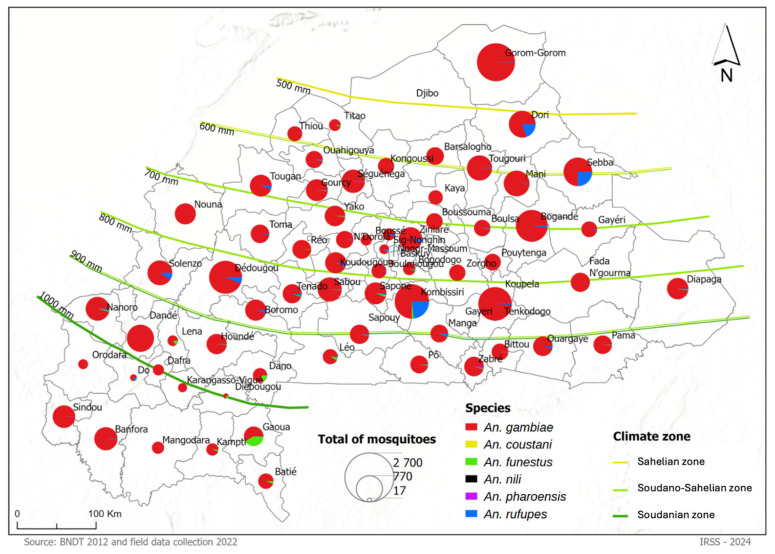
Spatial distribution and species composition of *Anopheles* mosquitoes per Health district in Burkina Faso, in 2022. Each pie chart represents the relative abundance of *Anopheles* species identified within a health district, with circle size proportional to the total number of specimens collected from September to December. The most prevalent species, *An. gambiae s.l.*, is represented in red, while other species (*An. funestus*, *An. nili*, *An. pharoensis*, *An. rufipes*, *An. coustani*) are shown by distinct colours as indicated in the legend. Green border lines indicate mean annual rainfall isolines (500–1000 mm) delineating the Sahelian, Soudano–Sahelian, and Soudanian climatic zones.

A total of 30,521 *Anopheles* specimens were collected between September and December 2022 across the 67 health districts spread in the three climatic zones of Burkina Faso: Sahelian, Sudano-Sahelian, and Soudanian. *Anopheles gambiae s.l.* was the most dominant species, representing 94.38% (28,820/30,521) of the overall *Anopheles* mosquito specimens collected (Figure 2). Other collected *Anopheles* species included *An. rufipes* (4.06%; 1239/30,521), *An. funestus* (1.36%; 415/30,521), *An. pharoensis* (0.11%; 33/30,521), *An. nili* (0.04%; 12/30,521), and *An. coustani* (0.01%; 2/30,521) (Table 1).

The Sahelian zone was dominated by *An. gambiae s.l.*, accounting for 91.85% (4743/5164) of all collected *Anopheles* mosquitoes. *Anopheles rufipes* was the secondary species, representing 8.03% (415/5164), while *An. funestus*, *An. pharoensis*, and *An. coustani* occurred at very low frequencies (<1%) (Table S1). Species composition remained relatively stable over time, with *An. gambiae s.l.* consistently exceeding 85% from September to December. Some spatial variability was observed, as *An. rufipes* reached higher proportions in specific districts, suggesting local environmental conditions may favour this species. 

In the Sudano-Sahelian zone, *An. gambiae s.l.* was the predominant species, accounting for 95.19% (17,602/18,492) of the collected anophelines, followed by *An. rufipes* at 4.1% (751/18,492), while *An. coustani* and *An. funestus* were detected at low frequencies. The dominance of *An. gambiae s.l.* was consistent across most districts and months, with a peak in October representing 95.89% (10,586/11,040) of all collections. However, some districts displayed notable temporal shifts toward the end of the rainy season. For example, in Kombissiri, *An. gambiae s.l.* decreased between October and November while *An. rufipes* increased during the same period. A similar pattern was observed in Solenzo, where *An. rufipes* rose in October before *An. gambiae s.l.* regained dominance in December (Table S1).

The anopheline species in the Soudanian zone was overwhelmingly dominated by the *An. gambiae* complex, which represented 94.32% (6457/6865) of the total anopheline specimens collected. The second most abundant species was *An. funestus* with 4.32% (301/865), while other species such as *An. rufipes* (1.06%), *An. pharoensis* (0.22%) and *An. nili* (0.01%) were significantly rarer.The dominance of the *An. gambiae* complex was consistent across most districts and throughout the sampling period, with monthly proportions generally exceeding 90%. However, *An. funestus* reached notable levels in certain districts. In Gaoua, this species accounted for 38.64% of captures in October and became the most prevalent species in December (66.67%). Similar increases were observed in Dano in November (46%) and in Léo in December (57.14%). These observations indicate that although the *An. gambiae* complex remains the primary vector in this ecological zone, *An. funestus* may locally assume greater importance during specific periods (Table S1).

### 3.2. Biodiversity Indices Across Climatic Zones and Collection Periods

**Figure 3:**
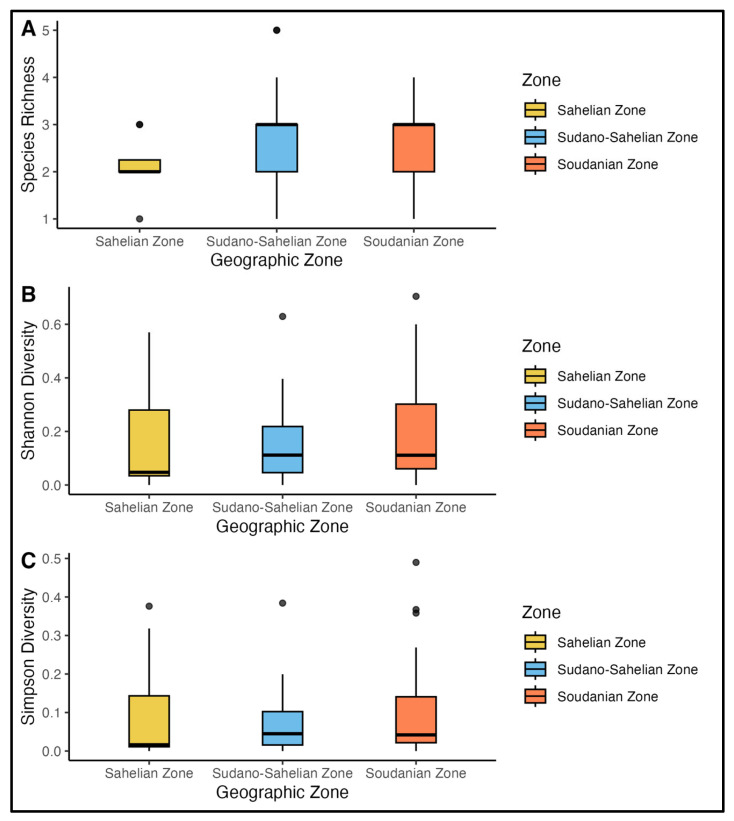
Variation in *Anopheles* species diversity across climatic zones of Burkina Faso, in 2022. Boxplots show differences in (**A**) species richness, (**B**) Shannon diversity index (H′), and (**C**) Simpson diversity index (D) across the Sahelian, Sudan–Sahelian, and Soudanian zones. Each box represents the interquartile range with the median line, vertical lines indicating data variability outside the upper and lower quartiles, and dots representing outliers. Diversity indices were computed from *Anopheles* collections recorded between September and December 2022, illustrating spatial patterns of species diversity along climatic gradients.

Biodiversity patterns of *Anopheles* mosquitoes were assessed across the 67 health districts spread in the three climatic zones of Burkina Faso (September–December 2022) using species richness, Shannon (H′), and Simpson (D) diversity indices. Species richness quantified the number of *Anopheles* species per zone, while the Shannon and Simpson indices integrated both species abundance and evenness to describe population diversity and dominance patterns, revealing marked spatiotemporal variation driven by seasonal and environmental factors (Figure 3).

The arid Sahelian zone exhibited the lowest biodiversity with richness ranging from 1–3 species per district-period (Figure 3). This low diversity was particularly evident and most observed in Barsalogho, Kongoussi and Thiou where only one species was collected, resulting in Shannon and Simpson diversity indices of zero across all sampling periods. The highest diversity was recorded in Sebba during October (Richness = 3, Shannon = 0.85 and Simpson = 0.46), reflecting temporary habitat suitability during peak of rainfall (Table S2). Dori and Titao districts showed moderate diversity in October to November (Shannon = 0.78–0.53, Simpson = 0.41–0.51), but diversity reduced to zero by December as water dried up. This temporal decline reveals the significant environmental constraints in the region. Only *An. gambiae s.l.* (*An. coluzzii*) appears to survive in the few remaining permanent water sources, while other species cannot withstand the dry conditions. 

The transitional Sudano Sahelian zone displayed intermediate biodiversity with greater spatiotemporal heterogeneity. Richness varied widely (0–5 species) with Solenzo recording the highest exceptional diversity in October (richness = 5, Shannon = 1.14 and Simpson = 0.56) in this zone was comparable to Soudanian diversity (Table S2). In Kombissiri, species diversity remained relatively high across sampling periods. A notable peak in November (richness = 3, Shannon = 1.76, and Simpson = 0.77) confirms that the area’s variety of larval breeding sites provides a stable foundation for a multi-species mosquito population. The zone’s biodiversity reflects the interaction between seasonal water availability and competitive dynamics among *An. gambiae s.l.*

The humid Soudanian zone exhibited the highest and most stable biodiversity, with richness spanning 0–4 species but fewer zero diversity periods (Table S2). During the peak diversity period of October-November, several districts, notably Dano, Do, Gaoua and Kampti, consistently showed high species richness. This was accompanied by elevated Shannon diversity indices, surpassing 1.07 in Dano, 1.42 in Do and Kampti 0.50 during November, indicating a more balanced species distribution as reflected in their respective Simpson indices (0.53 and 0.51). Banfora and Zabré showed the widest temporal variation, with October peaks (richness = 3–4) collapsing to monospecific or absent collection by December, reflecting seasonal breeding site dynamics in floodplain areas. Gaoua’s sustained diversity into December (richness = 3, Shannon = 0.97, Simpson = 0.56) and suggested permanent water bodies supporting year-round multi-species communities, potentially including *An. funestus alongside An.gambiaes s.l.* (Table S2). The higher Simpson values in the zone indicate more equitable species proportions, consistent with greater habitat heterogeneity and reduced environmental stress compared to northern zones.

### 3.3. Modeling Simpson Diversity Across Climatic Zones

Simpson’s diversity index was calculated for the *Anopheles* population across the 67 health districts in Burkina Faso, spanning three climatic zones: Sahelian (*n* = 21 samples), Sudano-Sahelian (*n* = 96), and Soudanian (*n* = 57), for a total of 174 sampling observations representing site-level *Anopheles* assemblages from dry (November to December) and wet (September to October) seasons. Due to the high prevalence of structural zeros (37.4% overall, ranging 36.5–42.9% by zone) reflecting single species dominance, we applied beta regression on epsilon transformed values (ε = 5.8 × 10^−4^ to 1.1 × 10^−3^) using the glmmTMB package. For each zone, we compared null models against season effects via Akaike Information Criterion (AIC), with null models consistently preferred (Δ AIC = 1.2–2.0), indicating negligible seasonal influence on diversity. Globally, the null model outperformed the full zone × season interaction (AIC = −639 vs. −631, ΔAIC = 7.7), providing moderate evidence against climatic effects. The mean Simpson’s index was uniformly low across zones (0.094–0.132), with a global estimate of 0.110 (95% CI: 0.087–0.134), suggesting persistent dominance of few *Anopheles* species. Model diagnostics using the DHARMa package confirmed adequate fit at the zone level (dispersion *p* > 0.75; uniformity *p* > 0.01), though global uniformity showed minor deviation (*p* < 0.001), likely attributable to structural zero-inflation rather than model misspecification. This pattern underscores ecological homogeneity in *Anopheles* assemblages across Burkina Faso’s climatic gradients, with single species dominance (often *An.gambiae s.l.*) persisting irrespective of aridity or seasonality. Such low diversity may constrain malaria transmission dynamics and vector control efficacy, warranting species specific intervention over zone tailored strategies. 

### 3.4. Composition of Anopheles gambiae s.l. Species Across Climatic Zones 

**Figure 4:**
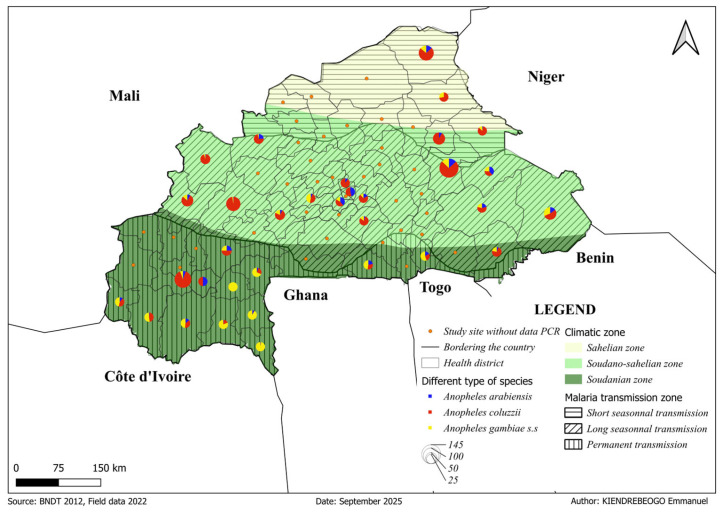
Molecular identification of *Anopheles gambiae s.l*. sibling species per climatic zones. Spatial distribution of *Anopheles gambiae s.l.* sibling species across climatic zones in Burkina Faso. Pie charts show the proportions of *An. arabiensis* (blue), *An. coluzzii* (red), and *An. gambiae s.s.* (yellow) identified by PCR in each selected health district. Circle size represents the number of specimens analyzed. Orange dots indicate sites without PCR data. Climatic zones are shown in shades of green, and hatching indicates malaria transmission intensity.

Polymerase chain reaction (PCR) analysis was conducted on 2026 *An. gambiae s.l* specimens collected between September and December across the three climatic zones in Burkina Faso (Table 1). The molecular identification revealed clear climatic structure of sibling species within the complex: *An. coluzzi*, *An. gambiae s.s.*, and *An. arabenssis* (Figure 4). The analyses revealed distinct species distributions across the climatic gradient. *Anopheles coluzzii* was the predominant species in both the Sahelian (74.9%, 143/191), and Sudano-Sahelian (71.0%, 632/890) zones (Table S3). In the Soudanian zone, *An. gambiae s.s.* accounted for most specimens (53.7%, 508/945), while *An. coluzzii* was less frequent (36.1%, 341/945). Only a small proportion of mosquitoes collected were *An. arabiensis*, which varied from 8.9% (17/191) in the Sahelian zone to 14.3% (127/890) in the Sudano-Sahelian zone (Table 1). A chi-square test showed that species distribution patterns were strongly dependent on climatic zone (χ^2^ = 353.86, df = 4, *p* < 0.00001), with a moderate effect size (Cramer’s V = 0.3). The analysis of residual provided clear detail, showing that *An. gambiae s.s.* was disproportionately abundant in the Soudanian zone (residual = 11.06) while being significantly underrepresented further north (residual = −4.05 in Sahelian, −9.52 in Sudano-Sahelian). PCR-based diversity indices showed all zones had richness = 3 species, but diversity increased southward. Analysis of diversity indices revealed a pronounced major latitudinal cline. Population evenness was highest in the Soudanian zone (Shannon = 0.93, Simpson = 0.57) due to the influential presence of *An. gambiae s.s.*, decreased in the transitional Sudano-Sahelian zone (Shannon = 0.80, Simpson = 0.45), and was most suppressed in the arid Sahelian zone (Shannon = 0.72, Simpson = 0.40). This pattern results from the increasing monopolisation of the mosquito population by *An. coluzzii* as conditions become drier.

## 4. Discussion

An entomological survey has been carried out for *Anopheles* specimen’s collection across Burkina Faso’s three major climatic zones. This study showed that a distinct ecological pattern dominated by *Anopheles gambiae* complex. Despite the country’s high climatic gradient from the arid Sahel in the north to the humid Soudanian zone in the south, mosquito populations exhibited remarkable structural uniformity. *Anopheles gambiae s.l.* accounted for over 94% of all *Anopheles* specimens, confirming its overwhelming predominance as the primary malaria vector in Burkina Faso. Molecular analyses further revealed clear geographic structure within the complex, with *An. coluzzii* dominating northern and central regions, while *An. gambiae s.s.* was more frequent in the humid southern areas. *Anopheles arabiensis*, though less common, was detected in all zones, suggesting broad ecological adaptability. These patterns are consistent with findings from Burkina Faso, Cameroon, Mali, and Niger [39,40,41] and align with previous national surveys [14,42]. Across the three climatic zones, several spatial and temporal variations complement the overall patterns reported in the results. In the Sahelian zone, although *An. gambiae s.l.* was largely dominant (91.85%), some districts showed higher proportions of *An. rufipes*, such as Dori (18.98%) and Sebba (24.41%), suggesting that local environmental conditions may favour this species. In the Sudano-Sahelian zone, the seasonal succession observed in districts like Kombissiri, where *An. gambiae s.l.* declined from 84% to 37% between October and November while *An. rufipes* increased from 15% to 47%, indicating that the end of the rainy season can shift species composition. Similarly, in Solenzo, *An. rufipes* temporarily rose to 44.66% before *An. gambiae s.l*. regained dominance in December. In the Sudanian zone, the generally high proportion of the *An. gambiae* complex (94.32%) was accompanied by notable peaks of *An. funestus* in specific districts, such as Gaoua (38.64% in October and 66.67% in December), highlighting the potential local importance of this species.

Our results confirmed previous studies indicating that malaria transmission in Burkina Faso is primarily driven by members of the *An. gambiae* complex and *An. funestus* [14,43]. Across sub-Saharan Africa, *An. gambiae s.l.* remains the principal malaria vector, with population peaks typically occurring during and immediately after the rainy season, when breeding sites are most abundant [44,45]. Seasonal shifts often involve increases in *An. funestus* populations in areas with permanent or vegetated water bodies, whereas mosquito densities in the Sahel decline sharply during the dry season due to the loss of larval habitats [45,46].

In contrast, in the East and Central African contexts, species composition and diversity vary substantially along climatic and altitudinal gradients [47,48]. The ecological uniformity observed in Burkina Faso likely reflects extensive anthropogenic landscape transformation. Agricultural expansion, irrigation schemes, and urban growth have produced stable aquatic habitats enabling ecologically flexible breeding sites for *An. coluzzii*. Moreover, the extended use of insecticide-based interventions may have selectively reduced populations of less resilient, habitat-specialist species, thereby promoting dominance of a few highly adaptable taxa [49].

A distinct biodiversity gradient was observed across Burkina Faso. The southern Soudanian zone showed the highest species richness and evenness, with the coexistence of *An. funestus*, *An. rufipes*, *An. pharoensis*, *An. nili*, and *An. coustani* alongside *An. gambiae s.l*. These species, although less abundant, may sustain residual malaria transmission during periods of low *An. gambiae* density, as reported in Ghana and Tanzania [50,51]. In contrast, the Sudano-Sahelian and Sahelian zones exhibited very low diversity (mean richness 1.0; Shannon and Simpson indices ≈ 0), consistent with the dominance of *An. gambiae s.l.* (91.8–95.2%). Similar declines in biodiversity with increasing aridity have been reported across West Africa [52]. These findings highlight the ecological and epidemiological implications of simplified vector populations. Low diversity may facilitate targeted control interventions but also increases vulnerability to environmental changes. Irrigation and peri-urban expansion could create new habitats favouring secondary species such as *An. rufipes*, which, although historically considered zoophilic, has been detected carrying *Plasmodium* in Burkina Faso [53]. Altogether, these patterns show that despite the overall dominance of *An. gambiae s.l.*, secondary species such as *An. rufipes* and *An. funestus* can play a more substantial role in specific districts and periods. This ecological configuration raises important operational considerations for malaria control. While the predominance of a single vector complex may facilitate targeted interventions focused on *An. gambiae s.l.*, such simplicity also increases vulnerability: any rise in insecticide resistance or behavioural shifts within this complex could undermine control efforts. Low species diversity therefore offers operational advantages but also strategic risks, highlighting the need for diversified and adaptive vector control approaches. The results showed that, despite the observed biodiversity gradient across climatic zones, mosquito population structure remains remarkably uniform. This pattern suggests a dominance constraint mechanism, in which strong environmental pressures and competition favour a few highly adaptable species. Members of the *An. gambiae* complex, with traits such as desiccation-resistant eggs and behavioural plasticity, thrive in both natural and human-modified habitats [43,54]. Human activities such as irrigation and agriculture strongly influence vector distribution and reinforce this ecological dominance [49]. These dynamics highlight the need for adapted control strategies, combining timely interventions before seasonal peaks with zone-specific approaches targeting dominant and emerging secondary vectors.

From a public health perspective, the uniformity of indoor collected *Anopheles* populations across Burkina Faso offers both advantages and risks. The dominance of *An. gambiae s.l.* supports continued use of LLINs and IRS, but also intensifies insecticide selection pressure, accelerating resistance. Given the higher frequency of pyrethroid resistance in *An. gambiae s.s.* from southern regions [43] a dual strategy is recommended: maintaining universal coverage while strengthening molecular surveillance. Integrating next-generation tools such as dual-active LLINs, larvicide, genetic control approaches, and environmental management will be essential to mitigate resistance and sustain long-term control effectiveness.

This study offers a broad overview of *Anopheles* diversity in Burkina Faso but is limited by its four-month sampling period, which did not capture full annual dynamics. Morphological identification may have underestimated cryptic species, and insecticide resistance was not evaluated. Future research should include year-round molecular monitoring and resistance testing, coupled with fine-scale environmental data to better understand local drivers of vector distribution. Our sampling approach, which relied mainly on indoor collections, may have overlooked exophilic or outdoor-biting mosquito species. Experimental studies on larval ecology and competition are also needed to clarify mechanisms sustaining *An. gambiae* dominance.

## 5. Conclusions

This study assessed *Anopheles* diversity and distribution across Burkina Faso, providing important ecological insights along the country’s major climatic zones. The persistence of *Anopheles* dominance across environmental gradients suggests that human activities such as irrigation, agriculture, and land-use change exert a stronger influence on vector composition than climate alone. A clear biodiversity gradient was detected, with secondary species occurring mainly in the southern zone. Although less abundant, these species may sustain residual transmission during periods of low *An. gambiae* density. Such ecological simplification challenges expectations of strong species turnover along environmental gradients and indicates a vector population that is highly adapted and relatively stable. The predominance of *An. gambiae s.l.* supports continued deployment of LLINs and IRS, but also underscores the need for enhanced molecular surveillance, development of genetic control tools, and adaptive management to mitigate insecticide resistance and environmental change. Overall, this work emphasises the need to monitor and design locally tailored, evidence-based interventions across the country’s ecological zones.

## Figures and Tables

**Table 1:** Species composition of *Anopheles gambiae s.l.* complex across climatic zones in Burkina Faso.

Climatic Zone	*An. gambiae s.s.* (%, n/N)	*An. coluzzii* (%, n/N)	*An. arabiensis* (%, n/N)	Total (%, N/N)
Sahelian	16.2% (31/191)	74.9% (143/191)	8.9% (17/191)	100% (191/191)
Sudano-Sahelian	14.7% (131/890)	71.0% (632/890)	14.3% (127/890)	100% (890/890)
Sudanian	53.8% (508/945)	36.1% (341/945)	10.2% (96/945)	100% (945/945)
Total	33.1% ( 670/2026)	55.1% (1116/2026)	11.8% (240/2026)	100% (2026/2026)

## Data Availability

The original contributions presented in this study are included in the article/Supplementary Material. Further inquiries can be directed to the corresponding author(s).

## Supplementary Materials

The following supporting information can be downloaded at: https://www.mdpi.com/article/10.3390/tropicalmed11010001/s1, Table S1: Species composition of *Anopheles* mosquitoes per Health district and collection period.; Table S2: *Anopheles* species diversity across climatic zones.; Table S3: *Anopheles gambiae s.l.* sibling species per climatic zone.
